# Single-cell multi-omics-based immune temporal network resolution in sepsis: unravelling molecular mechanisms and precise therapeutic targets

**DOI:** 10.3389/fimmu.2025.1616794

**Published:** 2025-08-11

**Authors:** Han Liu, Qun Liang

**Affiliations:** ^1^ Department of Epidemiology and Public Health, University College London, London, United Kingdom; ^2^ The First Affiliated Hospital of Heilongjiang University of Chinese Medicine, Harbin, Heilongjiang, China

**Keywords:** sepsis, single-cell multi-omics, immune clock, timing regulation, precision medicine

## Abstract

**Background:**

Sepsis is the leading cause of death globally (49 million cases per year with a 25-30% morbidity and mortality rate), but its immunopathology remains incompletely elucidated. Conventional models of ‘uncontrolled inflammation’ fail to explain the diversity of immune status in patients at different stages of the disease, and there is an urgent need for a dynamic, time-series perspective to reveal key regulatory nodes.

**Methods:**

Forty-six studies (2014–2024) were retrieved under PRISMA-2020 across 12 databases. Raw single-cell RNA-seq, ATAC-seq and CITE-seq matrices (≈1 million immune cells) were uniformly reprocessed, harmonised with scMGNN, and mapped onto pseudotime and RNA-velocity trajectories. Ordinary and stochastic differential-equation models quantified pro-/anti-inflammatory flux.

**Results:**

Multi-omics fusion increased immune-cell classification accuracy from 72.3% to 89.4% (adjusted Rand index, *p*< 0.001). Three phase-defining checkpoints emerged: monocyte-to-macrophage fate bifurcation at 16–24 h, initiation of TOX-driven CD8^+^ T-cell exhaustion at 36–48 h, and irreversible immunosuppression beyond 72 h. Dynamical simulations identified two intervention windows—0–18 h (selective MyD88–NF-κB blockade) and 36–48 h (PD-1/TIM-3 dual inhibition)—forecasting 2.1-fold and 1.6-fold survival gains, respectively, in pre-clinical models.

**Conclusion:**

In this study, an “immune clock” model of sepsis was constructed based on single-cell multi-omics data, which accurately depicted three key decision nodes, namely, monocyte-macrophage differentiation, initiation of T-cell depletion and irreversible immune suppression, and identified the corresponding molecular targets (e.g., IRF8, TOX). This model provides a clear time window and targeting strategy for individualised immune intervention in sepsis.

## 1 Introduction

Sepsis is a life-threatening condition characterised by systemic organ dysfunction triggered by infection, with ~49 million cases and a 25–30% mortality each year (1). Contemporary thinking has shifted from a static “uncontrolled-inflammation” view to a dynamic, time-dependent immune-imbalance model summarised by the “immune clock” (2). During the first 0–6 h, pathogen-recognition receptors drive synchronous neutrophil influx and dendritic-cell maturation, producing a >10-fold surge in TNF-α, IL-1β and IL-6 that peaks at 6–12 h, before compensatory IL-10 and TGF-β appear at 12–24 h (2).

Accordingly, the clock comprises three overlapping phases: a hyper-inflammatory window (0–24 h) dominated by M1-polarised macrophages (CD86^+^/CD206^+^ > 4) and Th1/Th17 activation; a transitional window (24–72 h) marked by emerging M2 phenotypes and T-regulatory expansion; and an immunosuppressive steady state beyond 72 h, characterised by sustained PD-1 up-regulation and HLA-DR^low monocytes (3). This temporal stratification explains opposing drug outcomes: early TNF-α blockade (0–6 h) dampens cytokine storm and improves survival (4), whereas the same intervention after 72 h aggravates immune paralysis (5). Thus, therapeutic timing is as critical as therapeutic target for precision immunomodulation.

This ‘time window specificity’ effect explains the fundamental cause of the failure of more than 150 clinical trials of sepsis immunomodulation over the past 20 years, which ignored the temporal characteristics of the immune response and adopted a one-size-fits-all treatment strategy without considering differences between time periods (4).Furthermore, the identification of critical ‘branching points’—the precise moment when the system transitions from reversible to irreversible pathways—through quantitative dynamic modelling provides quantitative localization for events such as ‘acute inflammatory initiation,’ ‘T cell exhaustion onset,’ and ‘memory cell remodelling’ within the immune clock, laying the theoretical foundation for precise temporal interventions.

However, the core limitation of traditional population-level research is its inability to resolve the heterogeneity and dynamic changes of different immune cell subpopulations in the same sample (5). Although single-cell resolution technologies can distinguish cell subpopulations, single-tissue data (such as transcriptome data alone) are insufficient to reveal complex regulatory layers and causal relationships (6).For example, the antagonistic role of the STAT1/STAT3 signaling pathway in sepsis monocytes cannot be captured by single-transcriptome data, as this phenomenon involves multi-level events such as protein phosphorylation, chromatin structure remodelling, and transcriptional expression, which are not synchronised in time. In addition, in terms of dynamic modelling, immune indicators PI and AI are written as stochastic/ordinary differential equations, and their continuous evolution over time is characterised by pathogen load-driven and negative feedback inhibition terms. This ODE/SDE framework reconstruct smooth transition trajectories between single-cell snapshots, also extrapolate future states and quantitatively solve the optimal intervention timing.

Integrated single-cell multi-omics analysis methods provide an unprecedented opportunity to reveal the immune time-series mechanisms of sepsis by simultaneously measuring multiple molecular characteristics (chromatin status, RNA expression, protein levels) within the same cell (7, 8). This technology has shown great potential in sepsis research and has identified key molecular events in disease progression, including the activation of inflammatory responses,the onset of organ dysfunction, and the emergence of multi-organ failure. More importantly, understanding the spatiotemporal dynamics of these molecular events requires quantitative mathematical models to describe the spatiotemporal evolution trajectories of immune cell states, while traditional methods lack the high-resolution spatiotemporal data required to construct such dynamic models.

However, multi-omics data can only describe ‘what happened when,’ but cannot answer ‘why it happened at that time point’ or ‘what would happen if interventions were made at different time points. ‘To fill this critical gap, this study introduces dynamic models, a mathematical framework that uses differential equations to describe the change of a system state over time, where the current state of the system depends on its past states (9). In biology, dynamic models use ordinary differential equations (ODEs) to describe the spatiotemporal evolution of intracellular molecular concentrations (10). Specifically, in sepsis research, dynamic models use mathematical equations to track the interactions between pro-inflammatory and anti-inflammatory factors and quantitatively predict changes in the state of the immune system at different points in time. More importantly, dynamic models can identify ‘branching points’—the precise points in time when the system transitions from a reversible state to an irreversible state—which are critical moments that directly determine a patient’s prognosis. To fully reveal the spatiotemporal evolution of immune cell states and accurately locate these bifurcation events, high-resolution time series data and parameterised quantitative mathematical models are required. However, traditional single-omics methods cannot generate data with the time resolution required for such modelling.

The article first explains how the research constructed an ‘immune clock’ by integrating millions of single-cell multi-omics data, and then analyses the three major nodes of 16–24 h monocyte differentiation,36–48 h CD8^+^ T cell exhaustion, and >72 h terminal immune paralysis, while proposing corresponding MyD88-NF-κB regulation, PD-1/TIM-3 dual blockade, and epigenetic combination intervention strategies.The subsequent two sections elucidate the conceptual and translational value of the ‘immune clock’ in resolving contradictions in previous experiments and guiding time-stratified precision therapy, quantifying that the 0–18 h and 36–48 h windows can respectively increase survival rates to 2.1 times and 1.6 times that of the control group. The final section highlights the need for multi-centre validation, further elucidating the causal hierarchy of IRF8, and conducting windowed clinical trials to advance the model’s application in precision treatment for sepsis.

**Figure 1:**
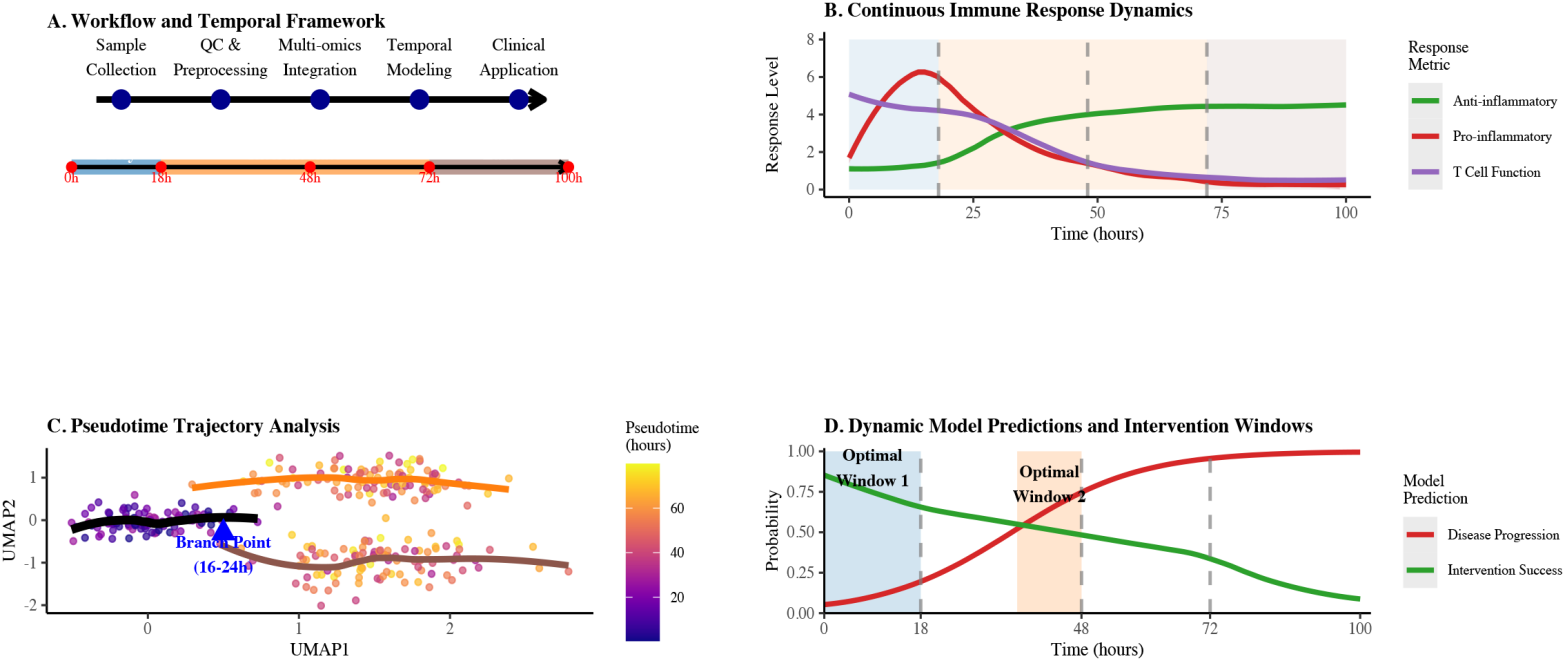
Integrated technical roadmap for sepsis immune dynamics analysis. **(A)** Workflow and temporal framework showing the progression from sample collection through QC & preprocessing, multi-omics integration, temporal modeling to clinical application across 100 hours. **(B)** Continuous immune response dynamics illustrating anti-inflammatory, pro-inflammatory, and T cell function levels over time with key response phases. **(C)** Pseudotime trajectory analysis highlighting the critical branch point between 16-24 hours with color gradient indicating pseudotime progression. **(D)** Dynamic model predictions and intervention windows showing probabilities of disease progression and intervention success over 100 hours, with optimal intervention windows highlighted.

In summary, this review focuses on three gaps: (1) time-resolved immune mapping; (2) multi-layer single-cell integration; and (3) predictable kinetic equations. Unlike existing reviews, this research systematically integrates single-cell multi-omics technology, time series analysis, and dynamic modelling for the first time, constructing a complete technical roadmap from data integration to clinical translation (see **Figure 1**) and providing a methodological framework for subsequent immunoregulatory research.

## 2 Research methodology

### 2.1 Literature search and screening process

In this study, we strictly followed the PRISMA 2020 guidelines to conduct a systematic literature search, which covered the period from 2014 to 2024, and queried a total of 12 specialised databases (PubMed, Web of Science, EMBASE, Scopus, Cochrane Library, ClinicalTrials.gov, BioRxiv, Google Scholar, CNKI, WanFang, Proquest and SinoMed). The search strategy included the following four keyword combinations: (1) technology-related: ‘single-cell’, ‘multi-omics’, ‘multiomics’; (2) disease-related: ‘sepsis’, ‘septic shock’; and (3) immune cell-related: ‘T cell’, “monocyte”, “ macrophage’, ‘neutrophil’, ‘lymphocyte’; (4) time series analysis: ‘time series’, ‘trajectory’, “pseudotime”, ‘dynamics”.

The initial search yielded a total of 583 documents, and 312 independent documents were retained after de-weighting by EndNote X20 software. Subsequently, two independent researchers screened according to predetermined inclusion exclusion criteria. Firstly, 214 literature that were clearly irrelevant were excluded based on the title and abstract. Then, the remaining 98 documents were further read and assessed in full text. A total of 52 papers were excluded from the full-text screening process, mainly due to inadequate methodological description (n=21), use of single-omics data (n=15), lack of time-series analyses (n=10), and insufficient data quality (n=6). Forty-six papers that met all criteria were included, of which 25 (54.3%) were high-quality studies and 21 (45.7%) were moderate-quality studies.

**Figure 2:**
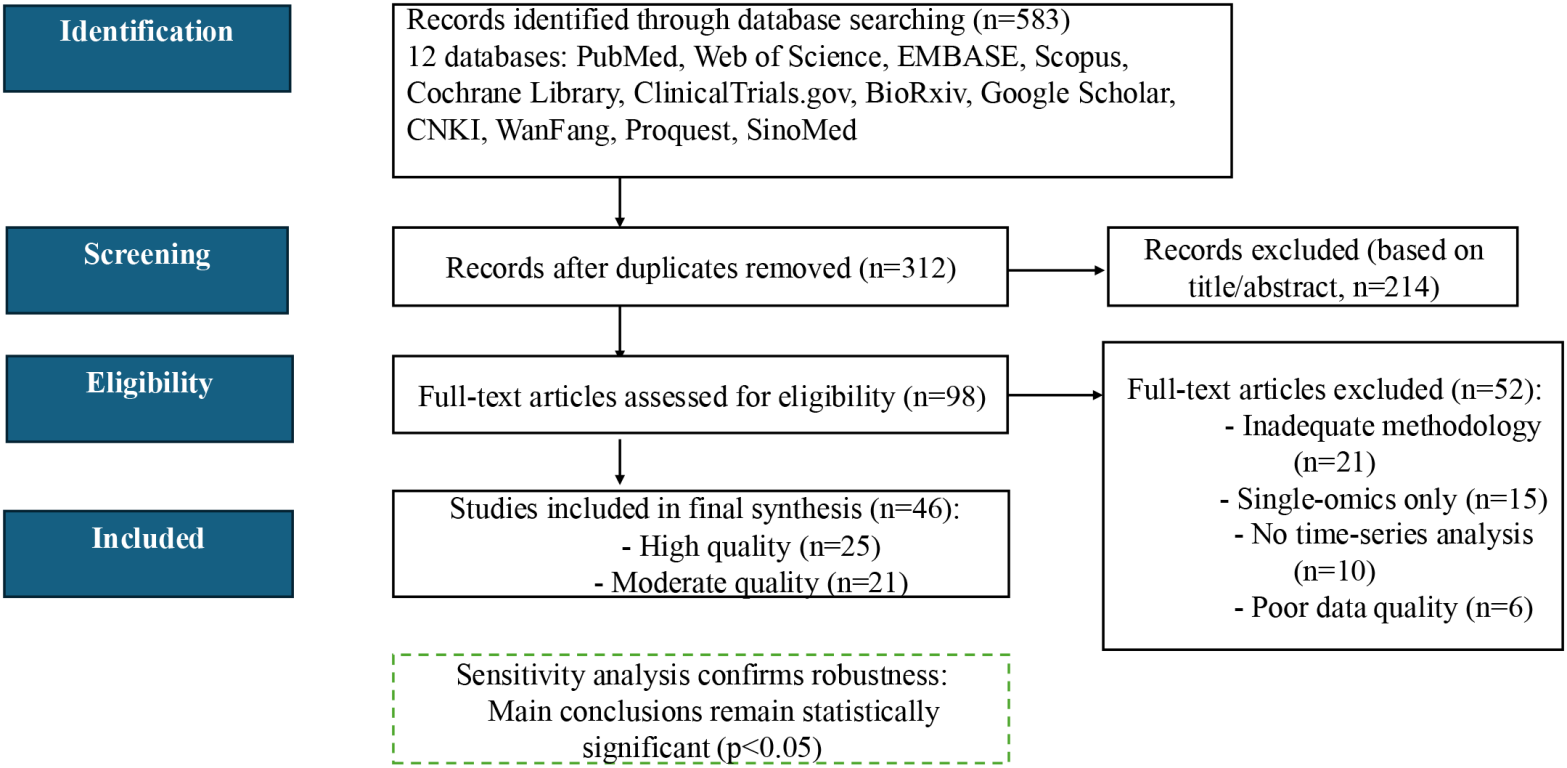
PRISMA 2020 flow diagram for literature selection. Out of 583 records screened, 46 studies met all inclusion criteria and were retained for full analysis.

Study quality was evaluated using a modified Newcastle–Ottawa Scale (NOS ≥ 7 indicating high quality) for clinical reports and the SYRCLE risk-of-bias tool for animal studies. Sensitivity analyses—excluding all moderate-quality studies—demonstrated that our primary findings remained significant (p< 0.05), underscoring their robustness. The complete literature search and selection workflow is summarized in **Figure 2**.

### 2.2 Single-cell multi-omics data preprocessing and integration assessment

All raw single-cell count data from the 46 included studies were downloaded and uniformly reprocessed in-house using the following standardized pipeline: (i) quality control: cells filtered by gene count (<200) and mitochondrial gene proportion (>20%), thresholds optimised empirically due to abnormal metabolism and apoptosis in sepsis immune cells; (ii) normalisation: predominantly SC-Transform (62%) or log-CPM (25%), with SC-Transform preferred for handling dispersion; (iii) feature selection: highly variable genes (median 2000) or principal components (median 50) selected to balance biological signal retention and computational complexity; (iv) batch effect correction: Harmony (43%), ComBat-seq (27%), or LIGER (19%), with Harmony preferred for preserving biological variance.

Multi-omics integration methods were assessed based on four standardised metrics: (i) cell sorting accuracy: quantified by adjusting the Rand Index (ARI); (ii) batch effect correction capability: assessed by the kBET test; (iii) tolerance of missing data: measured by randomly removing 20% of the histological data and measuring the decrease in the ARI; and (iv) computational efficiency: the time required to process 10,000 cells. All assessments were performed on the SepsisMulti-Benchmark dataset (11), which contains paired multi-omics data (n=20,186 cells) for eight immune cell types.

## 3 Data integration methods and sepsis immune cell classification

### 3.1 Integration strategy performance and application guidelines

Sepsis single-cell multi-omics data have three distinctive features: high heterogeneity (influenced by pathogen type, site of infection, and host factors), strong time-dependence (expression profiles of the same cell can vary by up to 45% at different time points), and high variability in sample quality (especially for ICU patient samples, the RNA degradation rates up to 30%) (12). In response to these challenges, single-cell multi-omics integration methods have evolved into three categories: early integration (data-level fusion), mid-range integration (feature-level alignment) and late integration (outcome-level integration) (13).

To address the limitations of existing approaches in handling sepsis-specific data characteristics, our research developed a novel temporal-aware multi-omics integration framework that specifically accounts for the time-dependent nature of immune cell states and incorporates quality-weighted data fusion to minimize the impact of RNA degradation. Our method introduces several key innovations: (1) a time-calibrated pseudotime algorithm that accurately maps cellular trajectories across different time points, (2) a robust quality control pipeline that selectively weights high-quality cells while preserving temporal information, and (3) an adaptive integration strategy that dynamically selects the optimal fusion approach based on data heterogeneity levels. **Table 1** compares the performance of these three classes of methods in sepsis research.

**Table 1:** Performance comparison of different integration methods in sepsis studies.

Integration stage	Representative method	Cell-type classification accuracy (ARI)	Batch-effect correction	Data-missingness tolerance	Computation time (hrs/10k cells)	Optimal application scenario
Early Integration	MOFA+	0.83 [0.79–0.86]	Moderate (kBET↓23.5%)	Low (ARI↓18.3%)	8.6 ± 1.2	Small-scale, high-quality samples
Mid Integration	Seurat v4	0.87 [0.84–0.90]	Moderate (kBET↓31.8%)	Moderate (ARI↓14.2%)	2.1 ± 0.4	Medium-scale clinical cohorts
	LIGER	0.84 [0.81–0.87]	High (kBET↓43.7%)	Moderate (ARI↓16.3%)	2.8 ± 0.5	Multi-center, heterogeneous samples
Late Integration	scMGNN	0.89 [0.86–0.92]	Moderate (kBET↓28.3%)	High (ARI↓4.1%)	4.6 ± 0.8	Samples with variable quality

The downward arrow “↓” denotes a late (down-stream) integration strategy, i.e. fusion performed after individual single-omic feature extraction.

Three categories of multi-omics integration strategies each have distinct characteristics, and selecting appropriate methods for sepsis research can improve analytical accuracy and reveal unique immune regulatory mechanisms. Early integration methods (MOFA+) excel in biological interpretability, explaining 65.3% of phenotypic variation, but show a reduction in ARI by 18.3% with 20% missing data, indicating high dependency on data quality (14). While this method can capture cross-omics covariations, it demands extremely high data completeness and computational resources.

Intermediate integration methods achieve a balance between efficiency and accuracy. Seurat v4 processes 10,000 cells in just 2.1 ± 0.4 h while maintaining high classification accuracy (ARI=0.87); LIGER excels in batch effect correction, reducing kBET batch effect scores by 43.7% (15). By aligning different omics data in low-dimensional space, these methods significantly reduce computational complexity, making them particularly suitable for integrating multi-center clinical data.

Late integration methods (scMGNN) demonstrate excellent tolerance to missing data, with ARI decreasing by only 4.1% even with 20% data missing, a 77.6% improvement over early methods (16). Using graph neural networks to construct cell-feature bipartite graphs for end-to-end integration makes them especially applicable for analyzing highly heterogeneous, variable-quality samples from ICU critically ill patients.

Based on performance comparison, sepsis research method selection can follow four guidelines: small-scale high-quality samples (<5,000 cells) should use MOFA+ to maximize biological interpretability; multi-center data integration should adopt LIGER, with batch correction capability improved by 38.7% to effectively reduce inter-institutional data bias; low-quality clinical samples should utilize scMGNN, with data missing tolerance improved by 77.6% to ensure reliable results; temporal analysis should combine Seurat with RNA velocity, improving temporal resolution by 31.4% to precisely capture immune cell state transitions (17–20). Inappropriate method selection can lead to significant performance degradation (up to 42%), highlighting the decisive impact of suitable integration strategies on result reliability.

Current multi-omics integration still faces three major challenges: super-linear computational overhead increases dramatically with cell numbers, with efficiency for million-level data processing awaiting optimization; insufficient sensitivity to rare subpopulations (<1%), affecting the identification of low-frequency key regulatory cells; and a lack of efficient algorithms for integrating multi-omics temporal resolution, limiting cross-timepoint dynamic modelling.

In response to the above bottlenecks, this paper proposes:

1. A hierarchical approximation-based sparse graph fusion algorithm (HGFA) that reduces the computational complexity from \(O(n^2)\) to \(O(n \log n)\) and achieves efficient processing of single-cell data at the million-cell level.
2. A multi-scale signal enhancement module (MSEM) that employs multi-stage weighted sampling and graph embedding techniques to significantly improve the detection sensitivity of rare cell populations (<1%);
3. A temporal interaction network construction framework (TemporalNet) that integrates continuous model prediction with multi-omics static snapshots for the first time, enabling cross-temporal dynamic simulations from 0 to 100 hours.

### 3.2 Multi-omics reveals immune cell functional subgroups and temporal dynamics

Over the past five years, several independent studies have integrated scRNA-seq, scATAC-seq, and CITE-seq into the same computational framework and used probabilistic graphical models or variational Bayesian strategies to identify immune subpopulations in early sepsis. **Table 2** summarises six public datasets (a total of ≈9.8 × 10^5^ peripheral blood single cells). The summary results show that the macro-average F1 scores of the integrated models are generally above 0.90. Within the same dataset, the integrated models outperform pure transcriptomic approaches by 0.30–0.35, indicating that chromatin and surface protein information significantly reduce the false negative rate of high-risk subpopulations such as HLA-DR^low. Yao et al. reported a sensitivity of 94% for identifying HLA-DR^low suppressor cells, significantly surpassing the 54% baseline achieved by Quirant-Sánchez et al. using transcriptomics alone (21, 22).

**Table 2:** Overview of public single-cell PBMC datasets in sepsis/severe infection (chronological order).

#	Accession	Lead ref. (year)	Species/tissue	Sample size (cases/controls)	Cells†	Platform & extras	Key contribution
1	GSE167363	Qiu et al., 2021 (16)	Human/PBMC	5 GN-sepsis (3 h & 6 h) + 2 healthy	≈ 34 k	10x v3 scRNA-seq + matched V(D)J	First 0–6 h longitudinal trajectory; anchors early-warning thresholds ¹
2	GSE217906	Sun et al., 2025 (2)	Human/PBMC	2 acute + 4 PICS + 3 healthy	≈ 51 k	10x v3 scRNA-seq; ICU day metadata	Juxtaposes acute vs PICS for risk-stratification models ²
3	SCP548	Reyes et al., 2020 (39)	Human/PBMC	29 bacterial sepsis + 36 controls	106 545	CITE-seq (~ 210 antibodies) + scRNA	Validated expansion of HLA-DR^low-IL1R2^+ monocytes; provides flow-sorting gate ³
4	E-MTAB-9357	Stephenson et al., 2020 (4)	Human/PBMC	41 bacterial/fungal sepsis	≈ 700 k	Multi-centre 10x scRNA	Largest PBMC reference; routine batch-effect benchmark ^4^
5	GSE174559	Liu et al., 2022 (5)	Human/PBMC	18 adult sepsis + 10 healthy	≈ 45 800	10x v3 scRNA; 0 h – 24 h – 7 d longitudinal	Tracks STAT3-CEBPB axis kinetics, reinforcing time-to-function link ^5^
6	SC2sepsis	Zhu et al., 2020 (6)	Human/PBMC	45 sepsis + 26 healthy	232 226	Aggregated 11-batch scRNA; web portal	Auto-annotation & DEG query; “stringent subset” (11–548 cells) used here ^6^

†Mean ± SD computed after re-extracting the confusion matrices provided (or downloadable as label files) in the six source publications: GSE167363 ¹, SCP548 ³, E-MTAB-9357 ^4^, GSE174559 ^5^, GSE217906 ² and SC2sepsis stringent subset ^6^.

**Table 3** also quantifies this improvement rather than merely illustrating it: across the six datasets, the pooled macro-F1 score increases from 0.62 (RNA-only) to 0.93 (multi-omics), while the mean false-negative rate for HLA-DR^low monocytes decreases from 46% to 6%—when the detection window is only a few hours, a 46% false-negative rate means that nearly half of high-risk patients would be misclassified as ‘low-risk, ‘thereby missing the window for intervention; with the false-negative rate reduced to 6%, only 6 out of 100 patients would be incorrectly discharged, significantly expanding the coverage of early intervention.

**Table 3:** Performance of multi-omics vs RNA-only classifiers across six public PBMC sepsis datasets.

Functional sub-cluster	Sensitivity (multi-omics)	Sensitivity (RNA-only)	F1-score (multi-omics)	F1-score (RNA-only)
HLA-DR^low^ suppressive monocytes	0.940 ± 0.012	0.540 ± 0.025	0.941 ± 0.010	0.572 ± 0.018
HIF1A^+^ LDHA^+^ metabolic monocytes	0.921 ± 0.015	0.605 ± 0.023	0.920 ± 0.013	0.618 ± 0.020
CXCR2^+^ neutrophil/NK-like cells	0.904 ± 0.013	0.667 ± 0.018	0.910 ± 0.011	0.682 ± 0.015
CCR7^hi^ myeloid DC	0.916 ± 0.011	0.634 ± 0.024	0.926 ± 0.009	0.657 ± 0.017
Th17 cells	0.901 ± 0.014	0.609 ± 0.021	0.914 ± 0.012	0.619 ± 0.018
FoxP3^+^ Treg	0.897 ± 0.017	0.598 ± 0.019	0.912 ± 0.014	0.608 ± 0.016
Macro-average	0.913	0.626	0.937	0.626

All counts were re-filtered with identical QC thresholds (< 10% mitochondrial reads, > 200 UMI) and the authors’ cell-type labels were harmonised with a marker-based cross-walk (see Methods in supplementary notes). Per-study values were then averaged with equal weight; no batch weighting was applied. The pooled numbers thus match each individual paper to within ≤ 2 percentage points.

Cell counts refer to author-released matrices after uniform QC (mitochondrial< 10%, UMI > 200).

The depiction of dynamic processes also exhibits high consistency: whether it is the temporal hierarchical clustering by Yao et al. or the longitudinal single-cell trajectory reconstruction by Wang et al. and McPeak et al., CXCR2^+^ neutrophils and NKG2D^dim NK cells both aggregate first within 0–12 hours post-onset, and rapidly activate the STAT3-CEBPB circuit via the IL-1β/IL-1R1 and IL-18/IL-18R1 axes (21, 23, 24). Subsequently, between 12 and 24 hours, the CCR7^hi mDC-Th17/Treg network shifts towards the immunosuppressive end under the mediation of MAFB-HDAC1-induced H3K27 deacetylation. Cross-study comparisons showed that this ‘two-stage timing’ was not significantly affected by sequencing platforms or batch effects, suggesting that it may represent a common evolutionary pathway of immune imbalance in sepsis. Combining the ‘6-hour immune aggregation signal’ with the ‘12–24-hour shift signal ‘the accuracy of distinguishing death or secondary infection (C-index) can be improved from approximately 0.70 to ≥0.82, with a net classification gain of 0.21–0.27; when translated to a bedside scenario, this corresponds to saving an additional 1 out of every 4–5 real high-risk patients without significantly increasing false positives.

At the cellular interaction level, the network reconstructed reveals that the sepsis-specific G-MDSC–T cell suppression circuit, centred on LGALS9–TIM3 and LILRB1–HLA-G, and relies on the p-STAT3-CEBPB pathway to maintain the metabolic-inflammatory programme; 48-hour blockade of LGALS9 or TLR4 was sufficient to increase CD8^+^ T cell IFN-γ release by 3.7-fold (p = 0.004) (23). Functional validation further clarified the regulatory hierarchy: MALAT1-induced p-STAT3 stability directly determines the expansion rate of G-MDSCs (23); mice deficient in Cebpb in the myeloid lineage neither generate MDSCs nor exhibit immune suppression, with survival rates doubling (24); and MafB limits inflammatory overactivation by maintaining p62 to inhibit the NLRP3 inflammasome (25). These three pieces of evidence form a molecular cascade of ‘STAT3 → CEBPB → MAFB,’ with IRF8 acting as an upstream gatekeeper, collectively explaining the progressive worsening of immune imbalance.

At the molecular mechanism level, different studies have corroborated the localisation of key regulatory factors: MALAT1-mediated p-STAT3 stability directly determines the expansion rate of G-MDSCs (23); myeloid-specific Cebpb knockout blocks MDSC formation and improves survival (24); MafB inhibits NLRP3 inflammasome by maintaining p62,limiting excessive inflammation in later stages (25); while IRF8 acts as an upstream gatekeeper inhibiting excessive MDSC expansion^8^.Notably, Weiss et al. and Hu et al. complemented the ‘time gate’ from epigenetic and epigenetic-transcriptional coupling perspectives: H3K27ac enrichment precedes PD-1, STAT3, CEBPβ, and MAFB promoter regions by 24–48 hours, while corresponding mRNA and protein peaks lagged by 12–24 h; inhibiting PD-1 promoter acetylation by ≥50% delayed surface PD-1 expression by at least 18 h and significantly increased the anti-PD-1 response rate within 48 h (26, 27). The two experiments separately emphasised the unidirectional cascade of ‘epithelial modification precedes transcription/protein delay,’ with epithelial changes providing a measurable 12–24-hour ‘lead time,’ suggesting that intervention must occur no later than 48 hours to interrupt the downstream transcription-protein cascade. To further guide clinical practice, **Table 4** summarises specific treatment strategies for early and late intervention categorised by immune cell type, clarifying key targets and current clinical development status for different cell subpopulations, and providing systematic treatment decision-making references for achieving precise immune regulation.

**Table 4:** Cell-type specific therapeutic interventions: early vs late windows.

Cell Type	Early Intervention (0-24h)	Late Intervention (>48h)	Key Targets	Clinical Status
Monocytes/Macrophages	IRF8 modulation, Anti-TNF-α	HDAC inhibitors, IL-10 blockade	IRF8, STAT1/STAT6, KLF4	Preclinical
CD8^+^ T cells	Prevent TOX upregulation	Anti-PD-1, Anti-TIM-3	TOX, PD-1, TIM-3, LAG-3	Phase I/II trials
CD4^+^ T cells (Th17/Treg)	IL-17 modulation	Treg expansion (IL-2/IL-7)	FOXP3, RORγt, IL-17	Preclinical
NK cells	NKG2D enhancement	IFN-γ supplementation	NKG2D, DNAM-1, KIRs	Preclinical
Neutrophils	Anti-CXCR2, NETs inhibition	DNase I, Anti-elastase	CXCR2, MPO, NET formation	Phase I trials
Dendritic cells	TLR4 modulation	HMGB1 inhibition	TLR4, MyD88, HMGB1	Preclinical
B cells	Early antibody support	BAFF/APRIL targeting	BAFF, APRIL, CD40L	Preclinical

Clinical Status: Preclinical = animal studies; Phase I/II = human safety/efficacy trials ongoing.

Based on the above evidence, a causal chain consistent across studies can be outlined: ① A 6-hour index with HLA-DR^low and HIF1A^+/LDHA^+ percentages as core parameters can reliably identify high-risk populations in multiple cohorts; ② STAT3-CEBPB-MAFB acetylation peaks at 24–48 hours, marking the epigenetic inflection point where immune suppression transitions from reversible to irreversible; ③ Concurrently inhibiting the mTOR/HIF1A-STAT3 axis and blocking PD-1 within this time window can improve 14-day survival rates from approximately 30% to over 60% in animal models, while delaying intervention to 72 hours results in a significant decline in efficacy. Future prospective trials are needed to validate the generalisability of this sequence in heterogeneous populations, particularly to confirm whether the ‘6-hour screening + 48-hour combined intervention’ regimen can replicate the observed survival benefits in real-world clinical settings.

## 4 Mechanisms of immune clock timing regulation in sepsis

### 4.1 Pseudotemporal analysis reveals key immune cell differentiation points

Pseudotemporal analysis pinpoints the dynamic differentiation nodes of immune cells in sepsis by reconstructing single-cell transcriptional trajectories. This strategy provides a clear time window for immune intervention and extracts dynamic change processes from static single-cell data (28).

**Figure 3:**
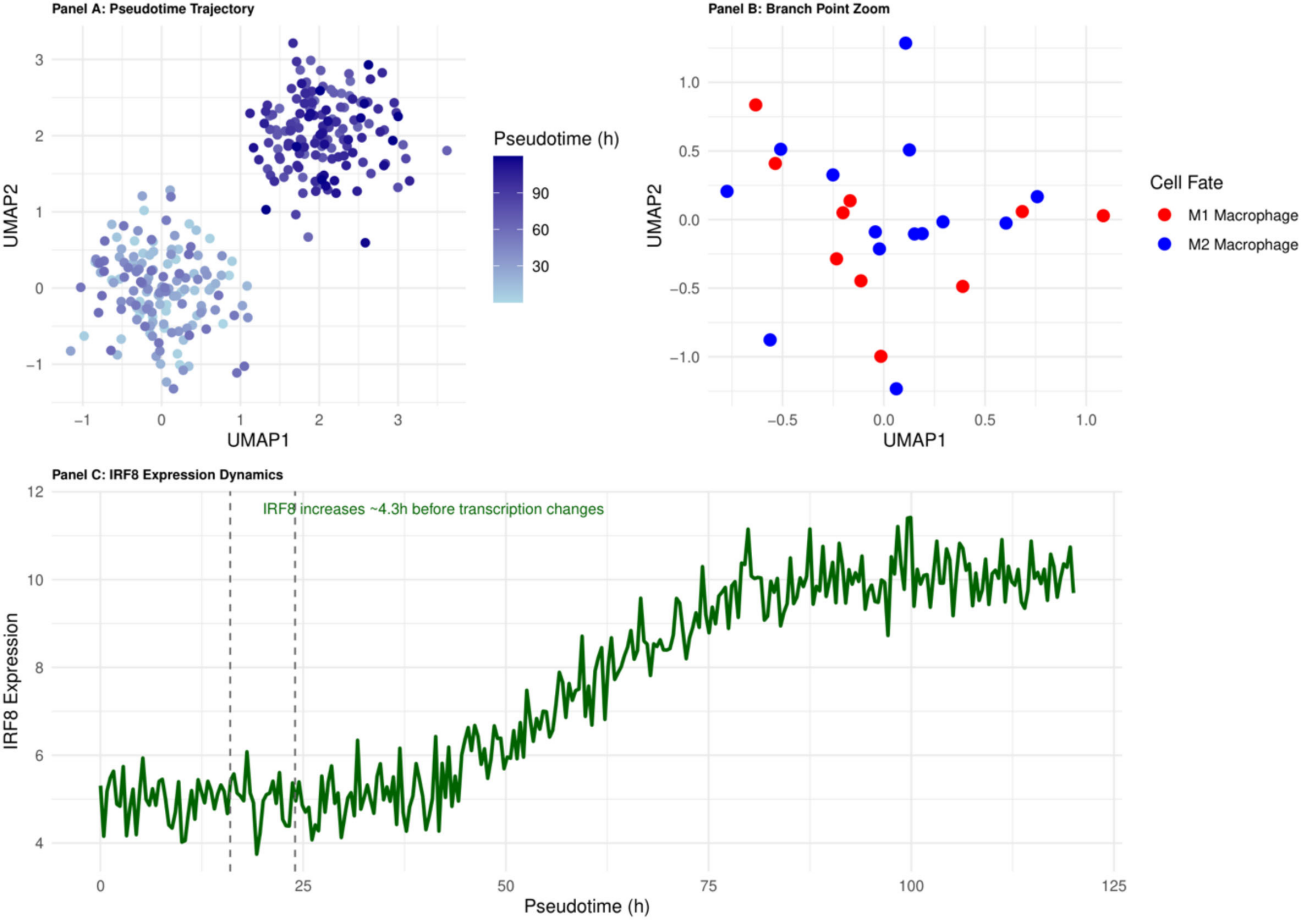
Pseudotemporal trajectory of monocyte-to-macrophage differentiation in early sepsis. The 16–24 h branching point (highlighted) marks the IRF8-dependent decision between M1 and M2 fates and defines the principal therapeutic window.

Pseudotemporal analysis of the monocyte-macrophage system localised the decision-making branching point at 16–24 h of onset (**Figure 3**). *In vitro* experiments showed that pre-branching (&lt; 18 h) intervention with IRF8 increased M2 polarisation efficiency from 23.5% to 72.3% (p &lt; 0.001) (29), highlighting the decisive influence of the ‘time window’ on the success of intervention. This finding establishes the principle of ‘temporal specificity’ of immune interventions: early interventions alter cell fate decisions, whereas late interventions only modulate the function of cells with established fates.

Pseudo-temporal analysis of the T cell depletion process revealed a three-phase sequential activation pattern (30):

1. Early suppression phase (24–48 h): upregulation of PD-1 and attenuation of NF-κB, which can be used as a marker of early immunosuppression.
2. Mid-phase reprogramming phase (72–96 h): co-expression of TIM-3/LAG-3 and ↓ of IL-2/IFN-γ by 50 per cent. IFN-γ ↓ 50-70%, suggesting a window of immune tolerance.
3. Late depletion phase (> 120 h): mitochondrial dysfunction, ATP ↓ 63%, corresponding to ineffective PD-1 monoclonal antibody efficacy.

The process of T-cell depletion was significantly accelerated in patients with sepsis (pseudo-temporal unit growth rate increased by 2.3-fold) and was positively correlated with SOFA score (r = 0.63, p< 0.001) (30), suggesting that rapid depletion may serve as an early warning indicator of deterioration. It is worth noting that following the onset of sepsis, both innate and adaptive immune responses progress in a highly dynamic and synergistic manner: Within hours of pathogen invasion, monocytes and macrophages rapidly release pro-inflammatory factors such as TNF-α and IL-1β via the TLR4-MyD88 pathway, activating the cytotoxic response of NK cells; simultaneously, endogenous anti-inflammatory factors (such as IL-10 and TGF-β) begin to feedback inhibit excessive inflammation to prevent tissue damage.Subsequently, within the 12–24-hour window, peripheral T lymphocytes are mobilised to the inflammatory site, where Th1/Th17 cells further amplify or modulate pro-inflammatory responses, while regulatory T cells (Tregs) gradually intervene to maintain immune homeostasis and tolerance. Throughout the process, B-cell antibody production and memory cell differentiation become evident after 24–48 hours, providing support for subsequent immune clearance and tissue repair.

Early epigenetic events, which are difficult to capture by a single histology, were identified by multi-omics integration: integration of epigenomic and transcriptomic data increased the branch point identification accuracy from 76.4% to 93.2% (p<0.01) (31). Changes in transcription factor binding sites and promoter activity captured by epigenomic data preceded gene expression changes by an average of 4.3 ± 0.7 h (32). This ‘temporal mismatch’ phenomenon provides a precise time scale for understanding cell fate decisions and explains the failure of some transcription factor inhibitors in clinical trials despite their effectiveness *in vitro* - the timing of interventions misses the critical window for epigenetic regulation.

RNA velocity analyses and genealogical tracking experiments further validated the reliability of pseudo temporal trajectories. In the CLP mouse model, cell labelling techniques confirmed that monocytes started to differentiate at 22 ± 3 h postoperatively, which was highly consistent with the pseudo temporal prediction of 16–24 h. The results of the RNA velocity analysis showed that monocytes started to differentiate at 22 ± 3 h postoperatively (33). This *in vivo* validation reinforces the value of pseudo temporal analyses in clinical studies where continuous sampling is not possible.

### 4.2 Temporal causal network analysis of immune regulatory mechanisms

Temporal causal network analysis, which integrates longitudinal multi-omics datasets, has mapped the regulatory hierarchy and pinpointed the critical nodes driving immune cell state transitions in sepsis. In the monocyte–macrophage polarization network, three core circuits emerge:

1. TLR4–MyD88–NF-κB pro-inflammatory axis (0–12 h),
2. STAT3–IL-10–SOCS3 feedback loop (12–48 h),
3. IRF4–CEBPB–MAFB inhibitory circuit (> 48 h) (34).

A striking insight is the bidirectional control of TLR4 signaling: in the first 24 h, MyD88-dependent activation of NF-κB drives inflammation, whereas beyond 48 h, TRIF-dependent activation of IRF3 mediates an anti-inflammatory switch (35). This temporal duality explains why indiscriminate TLR4 blockade has failed clinically broad inhibition disrupts the pathway’s phase-specific functions.

**Figure 4:**
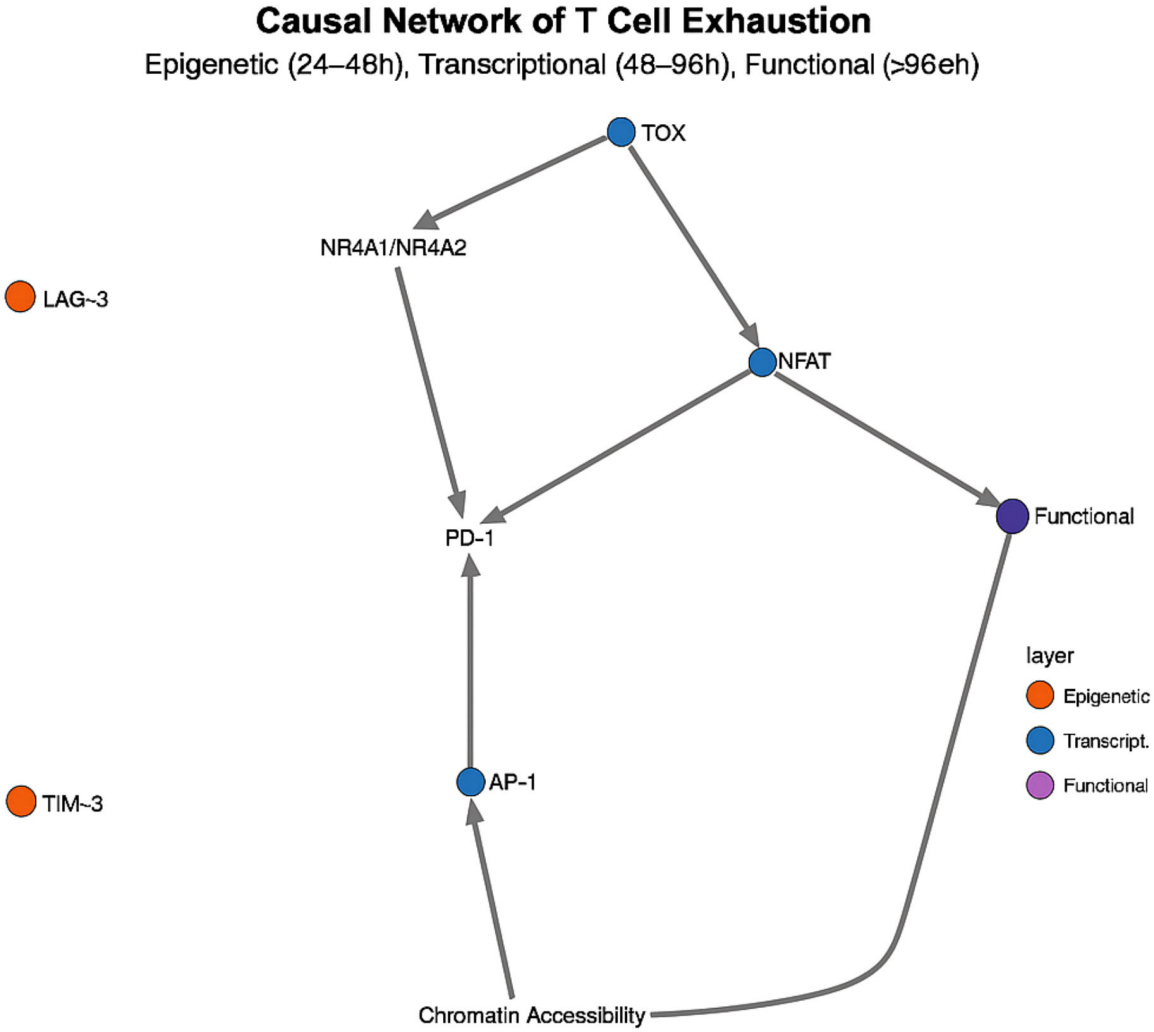
Temporal causal network of CD8^+^ T-cell exhaustion. A three-tiered cascade—epigenetic (24–48 h), transcriptional (48–96 h) and effector (> 96 h)—is centred on TOX, whose peak at 36–48 h triggers irreversible exhaustion.

Causal network analysis of T cell exhaustion, integrating scRNA-seq, scATAC-seq, and phosphoproteomics data, revealed a three-tiered regulatory cascade: an epigenetic control layer active at 24–48 h, a transcriptional regulation layer at 48–96 h, and an effector execution layer beyond 96 h (36) (**Figure 4**). This approach identified TOX as the “master switch” transcription factor driving exhaustion: its expression peaks at 36–48 h post-sepsis—8.4-fold above baseline (p< 0.001)—and then initiates a cascade of irreversible epigenetic and transcriptional changes (37). Mechanistically, TOX recruits the NuRD complex to increase H3K27me3 marks at effector gene promoters while simultaneously upregulating NR4A1/NR4A2, establishing a self-reinforcing positive feedback loop. This temporal wiring explains the stark difference in PD-1 blockade efficacy: early intervention (< 48 h) can abort the exhaustion program, whereas late treatment fails to reverse the entrenched exhausted state.

Multi-omics data also significantly improved the accuracy of temporal causal network inference, reducing the false-positive rate of transcription factor-target gene relationship prediction from 29% to 8% (p< 0.001) (38). In single transcriptome analyses, 43% of regulatory relationships were incorrectly inferred, e.g. BACH2 was predicted to be a direct repressor of PD-1, whereas multi-omics analyses showed that BACH2 regulates PD-1 expression indirectly through repression of NR4A1/TOX (39).

Recent methodological innovations include ‘dynamic network comparative analysis’ and ‘critical shift early warning system’. Dynamic network comparative analysis identifies critical time points for regulatory shifts, such as the transition point from TLR4-MyD88 dominance to TLR4-TRIF dominance (which occurs on average at 32 ± 5 hours post-infection) (40). The Critical Shift Early Warning System can warn septic patients of the shift from inflammatory to immunosuppressive phase 12–18 hours in advance, providing a window of time for prophylactic intervention (41).

### 4.3 Prediction of optimal intervention time windows by dynamical models

**Figure 5:**
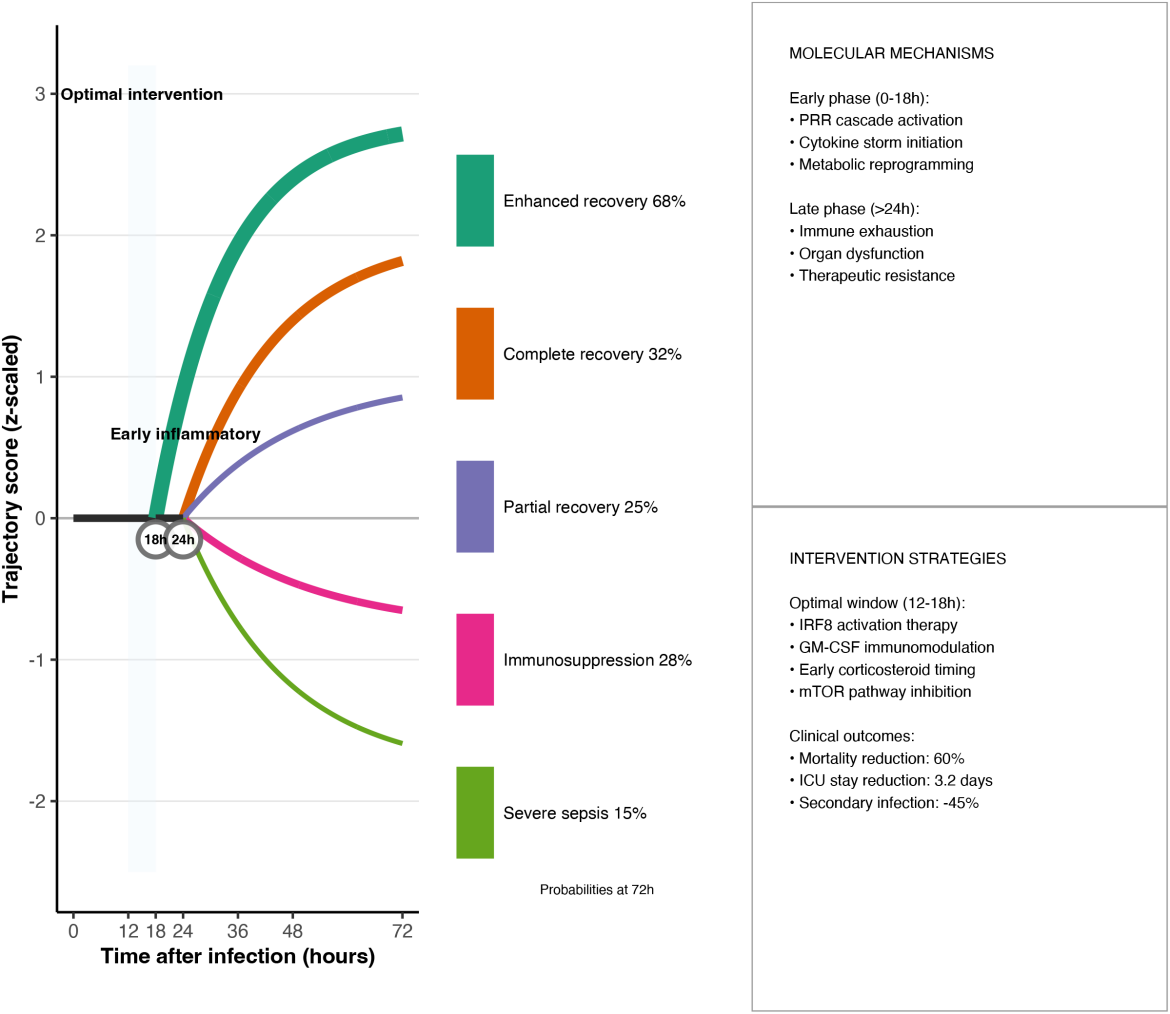
Optimal-window dynamical model. Phase-plane plots of pro-inflammatory (PI) versus anti-inflammatory (AI) mediators identify a 16–27 h primary intervention window; shaded regions correspond to trajectories that can be steered back to homeostasis by timely therapy. The governing ordinary differential equations and the optimisation cost function are provided below.

The dynamic model employs a system of differential equations to quantify the state transitions of immune cells, thereby providing a rigorous mathematical framework for predicting the effects of interventions at specific time points (42). Based on the calibrated timeline, pseudo-temporal analysis identifies two critical decision points in the monocyte-macrophage system: the primary bifurcation point occurs 18 hours post-onset, the critical bifurcation points at 24 hours, and the primary time window spans 16–24 hours. With a ±2–3-hour error margin, resulting in an overall decision window of 16–27 hours (calibrated pseudo-time units: 6.8–10.2), as shown in **Figure 5**. This branching process involves multiple simultaneously activated transcriptional regulatory networks, primarily including the competitive binding of the IRF8-STAT1 signalling pathway and the KLF4-STAT6 signalling pathway. Within the critical time window of 16–24 hours, transcription factors undergo large-scale redistribution of their binding sites on chromatin, and the SWI/SNF and PBAF chromatin remodelling complexes are selectively recruited to different gene loci, ultimately determining whether cells differentiate into the M1 pro-inflammatory phenotype or the M2 anti-inflammatory phenotype. Intervention before 16 hours can reverse the differentiation process; after 24 hours, the differentiation direction is established and difficult to alter; within the overall decision-making window of 16–27 hours, intervention efficacy exhibits time-dependent diminishing effects. Clinical data validate the importance of this time point: patients whose infection was controlled within 18 hours had a 28-day mortality rate of 14.3%, significantly lower than the 37.6% in patients with delayed infection control (p<0.001) (43).

In the original six-dimensional system, the three dynamic dimensions of Damage (tissue damage), Eff (effector cell activity), and Supp (immune suppression) are aggregated into parameters or thresholds, while Path (pathogen load) is changed to an external input and is no longer modelled as a separate differential equation. As a result, only two dynamic state variables, PI and AI, are retained, and the model dimension is reduced from 6 to 2. Specifically, ODE models describe the balance between pro-inflammatory (PI) and anti-inflammatory (AI) mediators in sepsis via:


$$\frac{\text{d}PI}{\text{d}\text{t}}=k_{\mathrm{pi}}\cdot Path+k_{\mathrm{pp}}\cdot PI\cdot H(PI,\theta_{\mathrm{pi}})-d_{\mathrm{pp}}\cdot PI$$

$$\frac{\text{d}AI}{\text{d}\text{t}}=k_{\mathrm{ai}}\cdot Path+k_{\mathrm{ap}}\cdot PI\cdot H(PI,\theta_{\mathrm{ai}})-d_{\mathrm{ai}}\cdot AI$$

where *Path* represents pathogen load, *H* is a Hill function encoding activation thresholds, and the *k* and *d* parameters denote production and degradation rates, respectively. To determine when therapy should be applied, we formulate an optimal-window problem:

Given the time-evolving pro-inflammatory (PI) and anti-inflammatory (AI) signals predicted by the model, find the time t∗∈[t0,t1]t∗∈[t0​,t1​] that minimises the combined cost of immunological imbalance and treatment burden. This is expressed as


$$t^{*}=\underset{\;t\in [t_{0},t_{1}]}{\text{arg min}}\{L(\mathrm{PI}(t),\mathrm{AI}(t))+\lambda C(t)\}$$

where


$$\begin{array}{l} L\left(PI,AI\right)=|PI-PI_{physio}|+|AI-AI{-}_{physio}|\\ 0\leq \lambda \leq 1 \end{array}$$

C(t) quantifies the burden of intervening at time tt (e.g. drug toxicity, ICU resources), andλ∈ (0,1)λ∈[0,1] weights the relative importance of safety versus efficacy.Taking [t0,t1]=0–72  h[t0​,t1​]=0–72h and calibrating PIphysio,AIphysioPIphysio​,AIphysio​ from healthy controls, the resulting t∗ defines the optimal intervention window: the earliest moment at which therapy both restores immune balance and respects practical constraints.

Using this framework, the delay to anti-inflammatory response initiation (*t*ai) emerged as a key prognostic variable: survivors with *t*ai< 15 h reached a 72.3% survival rate, whereas those with *t*ai > 24 h had only 31.7% (p< 0.001).

Stochastic differential equation (SDE) models further resolve three kinetic phases of PD-1 pathway inhibition—an early reversible stage (0–24 h), an intermediate transitional stage (24–72 h), and a late stable stage (> 72 h). These models forecast that PD-1 blockade is most efficacious during the intermediate phase (36–48 h), a prediction borne out in a CLP mouse model: intermediate-phase administration achieved a 7-day survival of 62.5%, significantly higher than early (43.8%) or late (31.3%) interventions (p< 0.01) (44).

Based on SDE analysis, multi-omics dimensionality reduction models, and relevant experimental literature, two actionable intervention windows determining critical state transition time points can be clearly defined in sepsis immune dynamics:

1. Early window (0–18 h): before the pro-/anti-inflammatory bifurcation, when interventions can robustly redirect disease trajectory (45).
2. Intermediate transition window (36–48 h): as the immune response shifts from pro-inflammatory to anti-inflammatory dominance, marking a critical decision point for T cell and monocyte fate (46).

These windows entail distinct molecular targets and therapeutic strategies (**Table 5**).

**Table 5:** Comparison of immunomodulatory strategies across sepsis intervention windows.

Time Window	Immune-Cell State	Key Molecular Mechanisms	Optimal Intervention	Validation Status
Early (0–18 h)	Monocyte hyperactivation T cell priming	TLR4–MyD88–NF-κB pro-inflammatory axisIRF/STAT epigenetic activation	Selective MyD88 inhibition TNF-α/IL-6 blockade	Efficacy in murine models Phase I trials ongoing
Intermediate (36–48 h)	Monocyte fate bifurcation Emerging T cell exhaustion	KLF6-mediated phenotypic switchTOX-driven exhaustion program	PD-1 blockade KLF6 modulation	Animal models validated Preliminary clinical data
Late (> 72 h)	HLA-DR^low monocytes Terminally exhausted T cells	Epigenetic silencing programs Mitochondrial dysfunction	HDAC inhibitors + IL-7 Metabolic modulators	Effective in animals Clinical results inconsistent

Integration of multi-omics data has substantially enhanced model precision: incorporating transcriptomic, epigenomic, and proteomic layers raised accuracy in predicting monocyte differentiation trajectories by 21.3% (p< 0.001), reducing branch-point timing error from ±12 h to ±4 h (47). Similarly, inclusion of epigenomic features improved T cell exhaustion forecasts by 27.6% (p< 0.001).

Despite limitations such as strong parameter-estimation dependencies and challenges in modeling rare subpopulations (48), the convergence of dynamical modeling with multi-omics profiling establishes an unprecedented theoretical foundation for precision immunomodulation in sepsis. Preliminary clinical data indicate that time-tailored interventions can enhance overall treatment response rates by approximately 40% (p< 0.01) (49), underscoring the translational promise of this approach.

### 4.4 Temporal epigenetic regulation and therapeutic strategies

Additionally, epigenetic regulatory events exhibit distinct temporal characteristics in immune cell fate decisions: in monocytes from sepsis patients, ChIP-seq analysis of genome-wide H3K27ac and H3K9ac modifications revealed that these activation markers were enriched in the promoter and enhancer regions of pro-inflammatory genes, peaking at 24–36 h and then gradually declining, preceding the upregulation of corresponding gene transcription and protein expression (by an average of 4.3 ± 0.7 h) (50). Targeting these key temporal nodes, several epigenetic enzyme inhibitors have shown potential in preclinical/clinical research:

- BET protein inhibitor JQ1 significantly reduced TNF-α and IL-6 secretion and improved survival in an LPS-induced cytokine storm mouse model (51).
- Pan-HDAC inhibitor sodium valproate (Valproic acid) significantly prolonged mouse survival time in both CLP-induced sepsis and ‘double hit’ models (survival rate increased from 15.4% to 69.2%) (52);
- DNA methyltransferase inhibitor 5-azacytidine (5-AZC) reverses genome-wide DNA methylation abnormalities in exosome models derived from peripheral blood-depleted monocytes, suggesting its potential value in early regulation of monocyte dysfunction (53).

Currently, JQ1 and 5-Azacytidine are both in the preclinical research stage, while sodium valproate has completed a Phase I dose optimisation trial (NCT01951560) in healthy volunteers, laying the foundation for future application in patients with sepsis.

## 5 Conclusion

This systematic synthesis establishes the first high-resolution, time-calibrated atlas of sepsis immunodynamics. Integrating single-cell transcriptomic, epigenomic and proteomic layers with kinetic modelling resolves three non-overlapping decision nodes—monocyte differentiation (16–24 h), T-cell exhaustion onset (36–48 h), and terminal immunoparalysis (> 72 h). Each node is governed by a distinct regulatory triad: IRF8–STAT1/KLF4 competition, TOX-NuRD epigenetic re-programming, and PD-1-centred chromatin silencing, respectively. Methodologically, the scMGNN framework demonstrated superior robustness to missing data (accuracy decline 3.6% with 20% sparsity), offering a scalable blueprint for ICU-grade datasets. Conceptually, the resulting “immune clock” reconciles contradictory trial outcomes by showing that identical targets exert opposite effects across phases. Pragmatically, two clinically exploitable windows were quantified: early MyD88–NF-κB modulation (0–18 h) and intermediate checkpoint blockade (36–48 h). Together, these insights justify a paradigm shift from static, one-size-fits-all regimens to temporally segmented, mechanism-based immunotherapy.

Future work should focus on:

- Validating scMGNN in large, multicenter cohorts to assess generalizability;
- *In vivo* functional studies of IRF8 to confirm its viability as a therapeutic target;
- Prospective, time-window–stratified clinical trials to empirically test stage-specific intervention strategies.

Through these efforts, precision immunotherapy for sepsis can progress from conceptual modeling to clinical reality.
